# Armodafinil as Monotherapy in Treating Narcolepsy with Cataplexy

**DOI:** 10.7759/cureus.76437

**Published:** 2024-12-26

**Authors:** Arshia Ahmed, Sara Tariq, Salman J Khan, Zia H Shah

**Affiliations:** 1 Internal Medicine, Guthrie Lourdes Hospital, Binghamton, USA; 2 Public Health, University of Massachusetts Amherst, Amherst, USA

**Keywords:** cataplexy, excessive daytime sleepiness, modafinil, narcolepsy, sodium oxybutate

## Abstract

In narcolepsy with cataplexy, sodium oxybate and the recently FDA-approved drug pitolisant are preferred medications. Armodafinil, a longer-acting, non-amphetamine stimulant, is often used in patients who have narcolepsy without cataplexy. It enhances alertness by increasing presynaptic dopamine transmission presynaptically, amplifying serotonin in the cerebral cortex, activating glutamatergic circuits, which may contribute to its vigilance-enhancing properties, and stimulating orexin activity. Armodafinil alone is approved for excessive daytime sleepiness (EDS) symptoms.

We report this case of narcolepsy with cataplexy, where armodafinil alone improved the symptoms of not only EDS but also cataplexy. It is known that sodium oxybate, through its gamma-aminobutyric acid type B (GABA-B) receptor agonist activity, helps in cataplexy and EDS. However, in some instances, like this patient, armodafinil alone improves the symptoms of cataplexy and maintains wakefulness.

## Introduction

Narcolepsy is a chronic neurological sleep disorder that affects the brain's ability to control wake-sleep cycles and is characterized by chronic, excessive attacks of drowsiness during the day. It is sometimes called excessive daytime sleepiness (EDS). Narcolepsy is recognized by symptoms of brief and sudden sleep attacks during any activity that may last around 15 minutes. Cataplexy is defined as a sudden loss of muscle tone affecting either a small specific muscle group or causing widespread muscle weakness, which may lead to collapsing and being unable to move, often triggered by emotions and sometimes mistaken for a seizure disorder. Sleep paralysis is a state of muscle weakness or flaccidity while fully conscious during the transition between sleep and wakefulness. Hypnagogic hallucinations are visual or auditory experiences that may occur before or during the sleep episode. The attacks are characterized by a rapid transition into rapid eye movement (REM) sleep, which is a key diagnostic criterion [1-3].

The disorder typically starts early in adulthood, affects both genders equally, and tends to stabilize in severity around the age of 30, with a prevalence ranging from 25 to 50 cases per 100,000 individuals. There is limited information on the incidence of narcolepsy with cataplexy. One study showed 0.74 per 100,000 person-years [4]. The search for underlying risk factors has not yet uncovered significant associations. The disorder causes considerable morbidity, resulting in difficulties in academic and social functioning. However, the condition is manageable and, fortunately, responds to treatment [4]. 

The hypothalamus produces a neuropeptide called hypocretin (orexin), which controls arousal and sleep states. In type 1 narcolepsy, which is associated with cataplexy, there is a loss of orexin-producing neurons, believed to be caused by an autoimmune process potentially triggered by an infection. The HLA haplotype DQB1*0602 is found in 95% of type 1 narcolepsy patients, though it is also present in about 20% of the general population without the condition. Narcolepsy type 2, which does not involve cataplexy, arises through unclear mechanisms. This type is thought to involve less destruction of hypocretin neurons and impaired signaling of orexin receptors [5,6]. 

Treatment for narcolepsy focuses on enhancing wakefulness and alertness through medications such as modafinil or long-acting armodafinil, along with stimulants that work by increasing the release or inhibits the reuptake of norepinephrine or dopamine. To reduce cataplexy episodes, drugs like sodium oxybate and venlafaxine are used, which act by modulating gamma-aminobutyric acid type B (GABA-B) receptors or histamine H3 receptors (H3Rs). Additionally, sodium oxybate is employed to address symptoms of disrupted nighttime sleep, sleep paralysis, and hypnagogic hallucinations [7-10].

## Case presentation

A 73-year-old female patient presented to the clinic for a follow-up for her narcolepsy and obstructive sleep apnea (OSA). In her early 30s, she started to develop episodes of sudden paralysis and loss of muscle tone triggered by emotional stimuli, usually laughter. Her first episode was at age 31 when she was laughing at the dinner table, and her face slumped into a soup bowl. The symptoms were frequent during her 30s when she suddenly lost her muscle tone and had near falls.

She remained fully conscious during these episodes, typically triggered by fatigue, laughter and, at times, emotional outbursts. The resolution of symptoms occurs spontaneously. Additionally, she experienced frequent nocturnal awakenings and fragmented sleep.

The patient's excessive daytime sleepiness and cataplexy had significantly impaired her occupational performance and social interactions. The cataplectic episodes were brief, lasting from a few seconds to a minute. Despite a comprehensive sleep history and clinical evaluation, these symptoms persisted, prompting further investigation.

A polysomnography study revealed disrupted nocturnal sleep architecture with frequent awakenings, consistent with narcolepsy. The multiple sleep latency test (MSLT) done in 2012 showed that the patient had four naps at two-hour intervals. She fell asleep in all the naps. She had shortened sleep onset latencies, with an average sleep latency of five minutes and two sleep-onset rapid eye movements (REM). The diagnosis of narcolepsy with cataplexy was confirmed given two sleep onset rapid eye movements (SOREMs).

The patient was initially started on methylphenidate for a few months, which was later changed to modafinil. She took modafinil for another couple of weeks, but she was still having symptoms later in the day and was switched to longer acting armodafinil. Armodafinil 250 mg once daily has not only improved her daytime alertness and overall functional capacity but completely resolved her cataplexy without any significant side effects. For the last 12 years, she has been without a relapse or any cataplexy episodes since the full dose of armodafinil.

## Discussion

Narcolepsy is a chronic neurological disorder characterized by excessive daytime sleepiness, cataplexy, hypnagogic hallucinations, and sleep paralysis. Cataplexy, a hallmark symptom, involves sudden loss of muscle tone triggered by strong emotions, often leading to falls or collapses [9]. Treatment typically focuses on managing both excessive daytime sleepiness and cataplexy [8]. Standard treatment for narcolepsy with cataplexy generally involves a combination of medications [10,11].

For excessive daytime sleepiness, stimulants such as modafinil, amphetamines, and methylphenidate are commonly used. Modafinil and armodafinil, a wake-promoting agent, are preferred for their lower abuse potential compared to traditional stimulants [10,11]. For cataplexy, medications such as sodium oxybate, antidepressants like selective serotonin reuptake inhibitors (SSRIs) or serotonin-norepinephrine reuptake inhibitors (SNRIs), and sometimes pitolisant are used. Sodium oxybate is especially effective but may not always be accessible or preferred by all patients [11-13].

**Figure 1:**
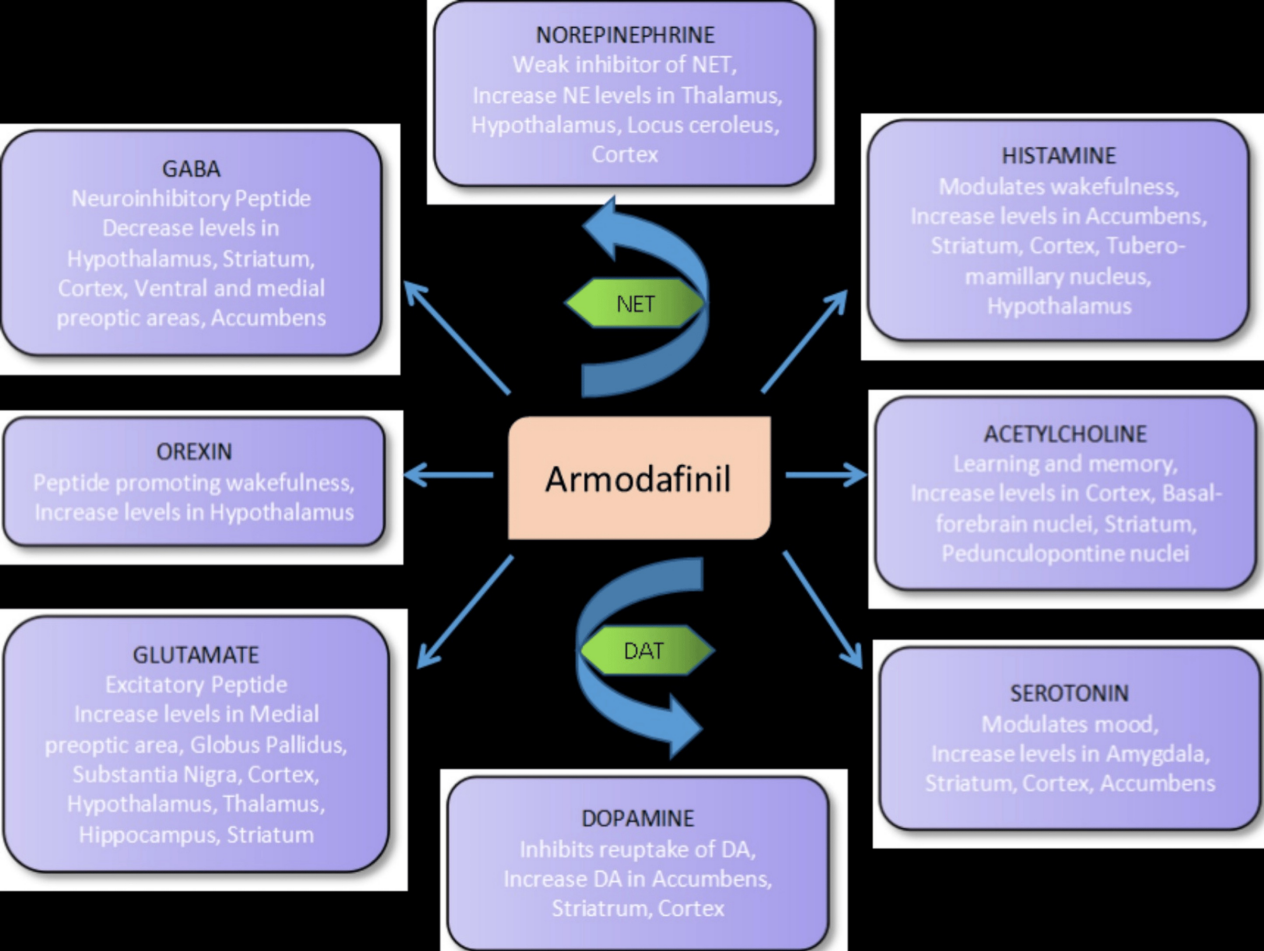
Mechanism of action of armodafinil. DAT: dopamine transporter; NET: norepinephrine transporter; DA: dopamine; NE: norepinephrine. Image created by Dr Sara Tariq.

Armodafinil binds to the dopamine reuptake pump, increasing dopamine levels and activating noradrenergic neurons in the locus coeruleus. The exact mechanism by which modafinil affects the noradrenergic system is still unclear, but it may stimulate downstream neurotransmission, potentially contributing to its cognitive-enhancing effects (Figure 1). Several studies have shown that modafinil increases extracellular serotonin in brain regions associated with cognition in a dose-dependent manner. Additionally, both acute and prolonged modafinil-induced wakefulness lead to long-term potentiation of glutaminergic and histaminergic synapses on orexin-producing neurons in the lateral hypothalamus [14].

The primary focus of this case report is to explore the efficacy of armodafinil alone in treating narcolepsy with cataplexy. Although armodafinil is primarily used for excessive daytime sleepiness, some studies suggest it may also have an impact on cataplexy.

In this case, the decision to use armodafinil as a monotherapy was based on: (a) Patient preference and tolerability: The patient preferred a simpler treatment regimen with fewer medications and reported good tolerability with modafinil, (b) Potential efficacy for cataplexy: Emerging evidence suggests armodafinil might exert some effect on cataplexy, although this is not well-established. This case provides additional insight into its potential role [15-16].

Existing literature generally recommends a combination of treatments for optimal management of narcolepsy with cataplexy. For example, sodium oxybate and antidepressants are frequently used to address cataplexy specifically, while modafinil or armodafinil are more commonly used for daytime sleepiness. The findings in this case align with limited reports suggesting that armodafinil might offer benefits beyond its primary indication, but more research is needed [15-18].

A study by Dauvilliers et al. in 2007 and a case report by da Silva Behrens et al. in 2014 indicated that modafinil could have a modest effect on cataplexy when used alongside other treatments [19,20]. However, this case demonstrates that modafinil alone might provide sufficient symptom control in some instances, at least in the short term. The specific mechanisms by which armodafinil affects cataplexy are not fully understood but may involve its impact on neurotransmitter systems, which are also implicated in cataplexy.

In this case, the patient showed significant improvements in daytime sleepiness and cataplexy with armodafinil alone. The reduction in cataplectic episodes from several times a month to approximately once in three months and then once in 30 years was noteworthy. This outcome is consistent with a few studies suggesting armodafinil may have some efficacy for cataplexy, although it is not universally recognized as a primary treatment for this symptom.

This case highlights the potential for modafinil to be considered as a monotherapy option in certain patients with narcolepsy and cataplexy, particularly when other treatments are contraindicated or not well-tolerated. While not a replacement for established therapies for cataplexy, modafinil’s effectiveness in managing both excessive daytime sleepiness and reducing the frequency of cataplexy episodes warrants further investigation.

## Conclusions

Armodafinil alone may offer a viable treatment option for narcolepsy with cataplexy in some patients, providing improvements in both daytime sleepiness and cataplectic episodes through its cognition-enhancing effect. This case report is limited by its single-patient focus but long-term follow-up. The long-term efficacy and safety of using modafinil or armodafinil alone for narcolepsy with cataplexy remains uncertain and requires larger, controlled studies. Future research should explore the role of armodafinil in the context of multi-modal treatment approaches and its potential benefits for a broader patient population, and further research is required to conclusively establish its efficacy and safety profile.
